# Transvection between nonallelic genomic positions in *Drosophila*

**DOI:** 10.1093/g3journal/jkad255

**Published:** 2023-11-09

**Authors:** Jacob A Blum, Michelle Wells, Zina Huxley-Reicher, Justine E Johnson, Jack R Bateman

**Affiliations:** Biology Department, 2 Polar Loop, Bowdoin College, Brunswick, ME 04011, USA; Biology Department, 2 Polar Loop, Bowdoin College, Brunswick, ME 04011, USA; Biology Department, 2 Polar Loop, Bowdoin College, Brunswick, ME 04011, USA; Biology Department, 2 Polar Loop, Bowdoin College, Brunswick, ME 04011, USA; Biology Department, 2 Polar Loop, Bowdoin College, Brunswick, ME 04011, USA

**Keywords:** transvection, long-range enhancer, somatic homolog pairing, promoter, chromatin, position effects

## Abstract

In *Drosophila*, pairing of maternal and paternal homologous chromosomes can permit *trans*-interactions between enhancers on one homolog and promoters on another, an example of transvection. Although *trans*-interactions have been observed at many loci in the *Drosophila* genome and in other organisms, the parameters that govern enhancer action in *trans* remain poorly understood. Using a transgenic reporter system, we asked whether enhancers and promoters at nonallelic, but nearby, genomic positions can communication in *trans*. Using one transgenic insertion carrying the synthetic enhancer *GMR* and another nearby insertion carrying the *hsp70* promoter driving a fluorescent reporter, we show that transgenes separated by 2.6 kb of linear distance can support enhancer action in *trans* at the 53F8 locus. Furthermore, transvection between the nonallelic insertions can be augmented by a small deletion flanking one insert, likely via changes to the paired configuration of the homologs. Subsequent analyses of other insertions in 53F8 that carry different transgenic sequences demonstrate that the capacity to support transvection between nonallelic sites varies greatly, suggesting that factors beyond the linear distance between insertion sites play an important role. Finally, analysis of transvection between nearby nonallelic sites at other genomic locations shows evidence of position effects, where one locus supported *GMR* action in *trans* over a linear distance of over 10 kb, whereas another locus showed no evidence of transvection over a span <200 bp. Overall, our data demonstrate that transvection between nonallelic sites represents a complex interplay between genomic context, interallelic distance, and promoter identity.

## Introduction

In *Drosophila* and other dipteran organisms, homologous chromosomes are closely paired from end to end in virtually all somatic cells (McKee 2004; Joyce *et al*. 2016). This chromosomal configuration can permit genetic regulatory regions to communicate between the 2 homologous chromosomes, a phenomenon known as transvection (Lewis 1954). Although examples of transvection and related *trans*-sensing phenomena continue to be uncovered and characterized in *Drosophila* and other organisms, the parameters that govern how genetic regulatory elements interact between 2 chromosomes in *trans* remain poorly understood.

**Fig. 1.**
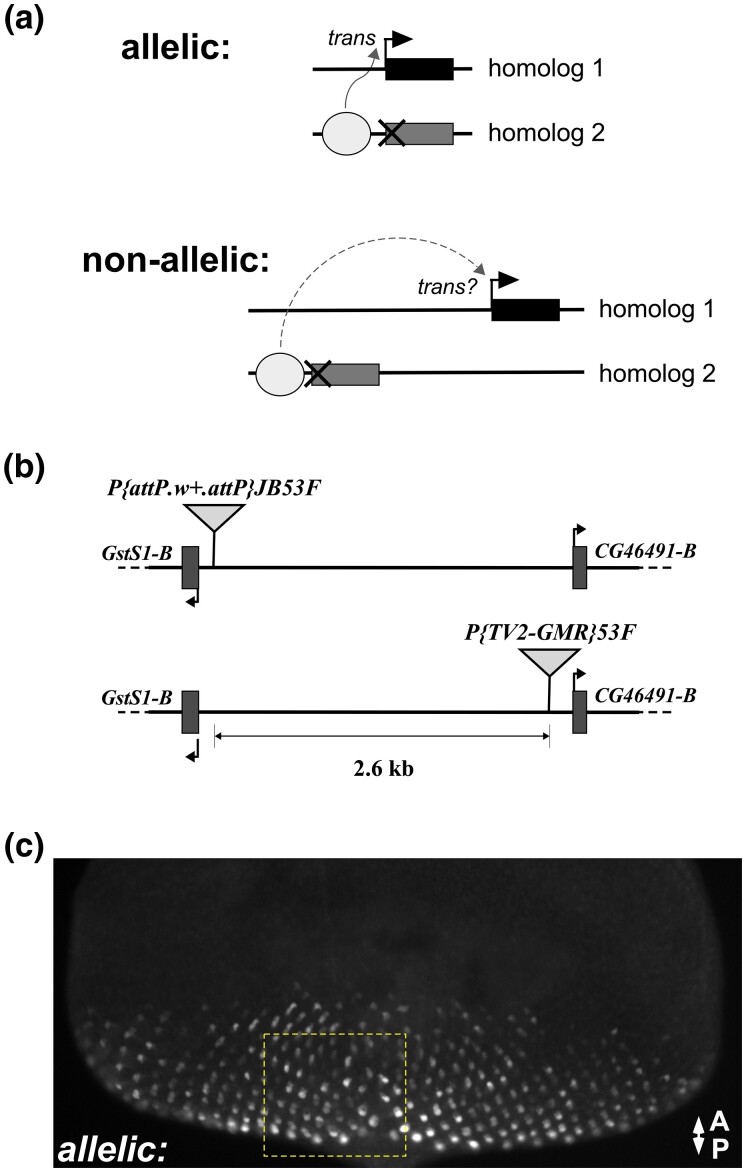
Transvection between nearby nonallelic positions. a) Schematic showing enhancer action in *trans* between paired homologous chromosomes, where one allele lacks an enhancer and the other allele lacks a promoter, at allelic positions (top), or nonallelic positions (bottom). Circle, enhancer; bent arrow, promoter; X, mutated or deleted promoter; rectangle, transcribed gene. b) Transgenic insertions used for prior transvection studies in polytene band 53F8. *P{attP.w+.attP}JB53F*, just upstream of the *GstS1-RB* TSS, is an RMCE landing site (Bateman and Wu 2008; Bateman *et al*. 2012), whereas *P{TV2-GMR}53F*, 2.6 kb distal to *P{attP.w+.attP}JB53F*, and just upstream of the *CG46491-RB* TSS, is based on the pWFL vector for transgene coplacement (Siegal and Hartl 1996; King *et al*. 2019). Both *GstS1* and *CG46491* are endogenously expressed in wild-type eye-antennal discs (Potier *et al*. 2014; Bateman and Johnson 2022). c) Third instar larval eye disc showing *GMR* acting in *trans* on the *hsp70* promoter driving *GFP* expression. Transgenes are inserted on homologous chromosomes at the *P{attP.w+.attP}JB53F* landing site. A and P, anterior and posterior. Dashed box indicates general field of view for higher-resolution images in Fig. 2.

In one form of transvection, an enhancer on one homolog can act in *trans* on a promoter on its corresponding homolog, stimulating transcription when the chromosomes are paired. Enhancer action in *trans* was first uncovered as a mechanism underlying intragenic complementation between specific types of mutant alleles. For example, if one allele lacks an enhancer (“Enhancerless”) and the other lacks a functional promoter (“Promoterless”), neither can support gene expression alone, but when paired, the remaining functional enhancer and promoter of the 2 alleles can interact in *trans* to achieve transcription (Fig. 1a; Lewis 1954; Geyer *et al*. 1990; Duncan 2002; Kennison and Southworth 2002). In most cases, alleles that carry a fully intact promoter in *cis* to a functional enhancer show weak enhancer action in *trans* (Martínez-Laborda *et al*. 1992; Casares *et al*. 1997; Gohl *et al*. 2008; Tian *et al*. 2019), or none at all (Geyer *et al*. 1990; Morris *et al*. 1998), suggesting that a promoter in *cis* to the functional enhancer is a preferred competitive target relative to a promoter in *trans*.

**Fig. 2.**
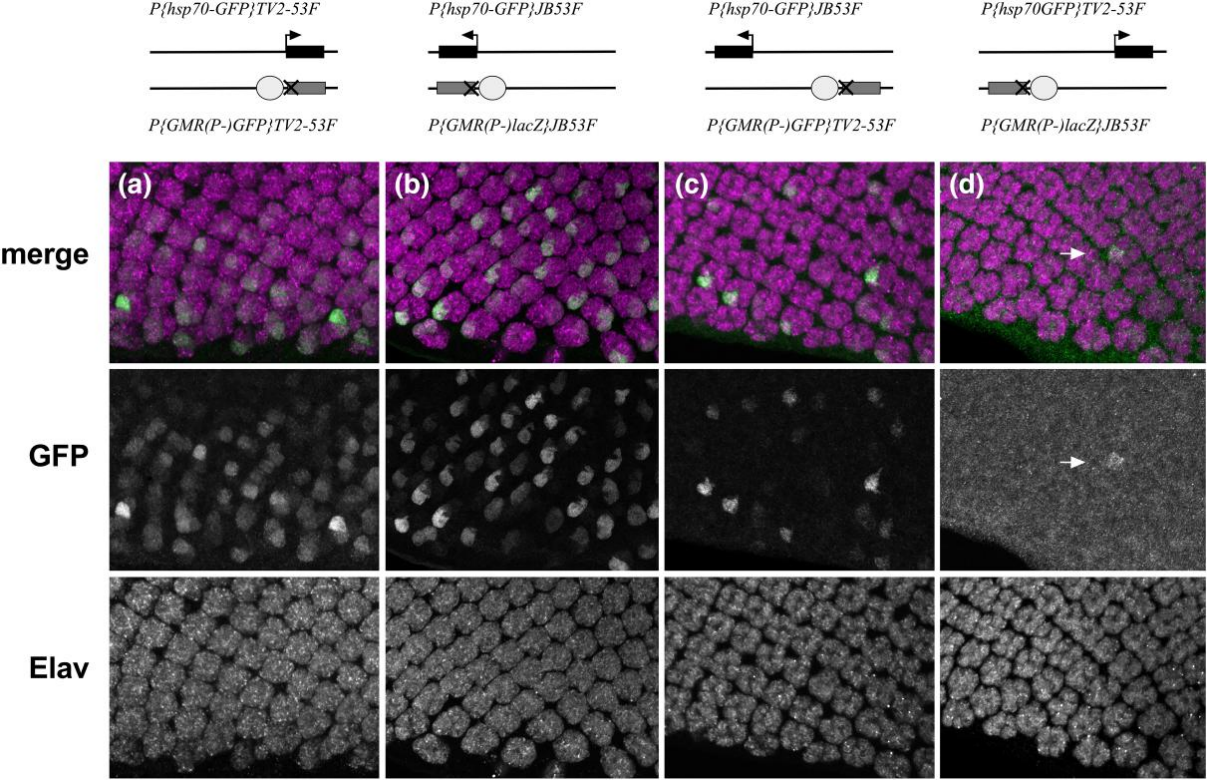
GMR acts in *trans* between nonallelic positions separated by 2.6 kb. Columns of images show merged or individual channels for staining with anti-GFP (indicative of transvection) or anti-Elav, a neural marker for all photoreceptors. Schematics above each image set show configurations of transgenes located at the 2 insertion sites shown in Fig. 1b. a and b) Enhancerless and Promoterless transgenes at allelic positions near *GstS1-RB* a) or near *CG46491-RB* b). c and d) Enhancerless and promoterless transgenes at nonallelic positions, with c and d carrying reverse configurations between the 2 insertion sites. Arrow in D, weak GFP signal in a single photoreceptor.

More recently, the study of enhancer action in *trans* has been bolstered by transgenic approaches, with site-specific integrases or recombinases allowing designed “alleles” of reporter genes to be inserted at identical positions in the genome (Chen *et al*. 2002; Bateman *et al*. 2012; Mellert and Truman 2012). In one approach, a P-element carrying recognition sequences for the integrase phiC31 is first randomly inserted in the genome; subsequently, the integrase attachment sites are used to precisely integrate Enhancerless and Promoterless fluorescent reporters that mimic alleles of endogenous genes where transvection has been observed (Bateman *et al*. 2012). Flies can then be generated where the Enhancerless transgene is carried on one homolog and the Promoterless transgene is carried at the precise allelic position on the other homolog, allowing the transgenes to pair and thereby permitting transvection between them (Fig. 1a). Transgenic systems have thus far been used to demonstrate that the *Drosophila* genome is generally permissive to transvection (Chen *et al*. 2002; Kravchuk *et al*. 2016; King *et al*. 2019), that the capacity to act in *trans* is common among enhancers (Mellert and Truman 2012; Blick *et al*. 2016), and that the incorporation of insulator sequences into transgenes can augment enhancer action in *trans* (Kravchenko *et al*. 2005; Schoborg *et al*. 2013; Lim *et al*. 2018; Piwko *et al*. 2019).

Since cytological evidence suggests that transvection in *Drosophila* ultimately relies on somatic homolog pairing, it may be reasonably assumed that productive *trans*-interactions between enhancers and promoters will be restricted to those that are encoded in allelic positions on homologous chromosomes. Indeed, disruption of homolog pairing by chromosomal rearrangement typically leads to loss of transvection and transvection-disrupting rearrangements are considered a form of experimental proof that bona fide pairing-dependent interactions are taking place between 2 alleles (Lewis 1954). Similarly, Enhancerless and Promoterless transgenes that are inserted into different locations in the genome will typically fail to support enhancer action in *trans*, presumably because they fail to pair with each other (Chen *et al*. 2002; Bateman *et al*. 2012; Mellert and Truman 2012), although the incorporation of sequences such as insulators and Polycomb Response Elements (PREs) into transgenes can sometimes cause insertions at nonhomologous positions to come together (Bantignies *et al*. 2003; Kravchenko *et al*. 2005). Notably, past experiments that tested interactions between nonallelic positions made use of transgenic insertions that are relatively far away from one another along the chromosome, or on different chromosomes altogether. However, it is as yet unclear whether Enhancerless and Promoterless transgenes that are inserted relatively close to one another on homologous chromosomes have the potential to productively interact (Fig. 1a). Exploring this question may reveal insight into how strictly homologous chromosomes align with one another.

Here we assess the capacity of Enhancerless and Promoterless transgenes that are inserted at nearby nonallelic positions to support enhancer action in *trans* using existing transgenic insertions from prior analyses of transvection (Bateman *et al*. 2012; King *et al*. 2019) and from large-scale screens for gene disruption (Bier *et al*. 1989; Huet *et al*. 2002; Bellen *et al*. 2004, 2011; Thibault *et al*. 2004). Our data show that transvection can be observed for transgenic insertions separated by at least 10 kb; however, in some cases, insertions separated by mere tens or hundreds of base pairs fail to support transvection, suggesting that factors such as genomic position and transgene identity have a major influence on productive *trans*-interactions between enhancers and promoters.

## Materials and methods

### Stocks and fly husbandry

Transgenic insertions used to assess transvection are described in Table 1. *H{Pdelta2-3}HoP8* is an X-chromosomal *hobo* insertion that constitutively expresses the P-element transposase. A multiply marked second chromosome carrying mutations in *aristaeless* (*al*), *dumpy* (*dp*), *brown* (*bw*), and *speck* (*sp*) was used to uncover male recombinants. All flies were cultured at 25°C on standard *Drosophila* cornmeal, yeast, sugar, and agar medium with *p*-hydroxybenzoic acid methyl ester as a mold inhibitor (Bateman *et al*. 2012).

**Table 1.** Insertions used in this study.

Insert	Polytene position	Genomic position*^a^*	Strand	References
*P{attP.w + .attP}JB53F^b^*	53F8	2R:17,097,510	+	Bateman and Wu (2008)
*P{TV2-GMR}53F^c^*	53F8	2R:17,100,154	−	King *et al*. (2019)
*P{TV2-GMR}42A^c^*	42A13	2R:6,214,221	+	King *et al*. (2019)
*P{TV2-GMR}37C^c^*	37C5	2L:19,158,447	−	King *et al*. (2019)
*P{lacW}GstS1[k11405]*	53F8	2R:17,097,378	+	Bier *et al*. (1989); Bellen *et al*. (2004)
*P{lacW}GstS1[k08805]*	53F8	2R:17,097,399	+	Bier *et al*. (1989); Bellen *et al*. (2004)
*P{lacW}GstS1[k09854]*	53F8	2R:17,097,454	+	Bier *et al*. (1989); Bellen *et al*. (2004)
*P{lacW}GstS1[k11301]*	53F8	2R:17,097,505	+	Bier *et al*. (1989); Bellen *et al*. (2004)
*P{lacW}GstS1[k10815]*	53F8	2R:17,097,631	+	Bier *et al*. (1989); Bellen *et al*. (2004)
*PBac{RB}CG30431[e01618]*	42A13	2R:6,203,346	+	Thibault *et al*. (2004)
*P{lacW}Ttc7[k15603]*	42A13	2R:6,214,072	+	Bier *et al*. (1989); Bellen *et al*. (2004)
*P{Epgy2}EY20090*	42A13	2R:6,214,143	−	Bellen *et al*. (2011)
*P{wHy}Ttc7[DG04810]*	42A13	2R:6,214,285	+	Huet *et al*. (2002)
*P{Epgy2}bin3[EY11308]*	42A13	2R:6,214,574	+	Bellen *et al*. (2011)
*P{Epgy2}bin3[EY09582]*	42A13	2R:6,214,695	−	Bellen *et al*. (2004)
*P{XP}brat[d11404]*	37C5	2L:19,158,258	−	Thibault *et al*. (2004)
*P{Epgy2}brat[EY01093]*	37C5	2L:19,158,440	+	Bellen *et al*. (2004)
*P{XP}brat[d08172]*	37C5	2L:19,161,727	+	Thibault *et al*. (2004)

^*a*^Insertion positions according to Release 6 of the *Drosophila* genome (dm6).

^*b*^Landing site for phiC31-mediated cassette exchange. The original insertion is marked with *mini-white*; Enhancerless and Promoterless GMR constructs were subsequently inserted using phiC31 integrase, which removes *mini-white* from the genome (Bateman *et al*. 2012).

^*c*^Insertion based on the pWFL vector for transgene coplacement (Siegal and Hartl 1996); Enhancerless and Promoterless GMR constructs are derived using Cre and FLP, respectively (King *et al*. 2019).

### Screen for male recombinants and flanking deletions

**Fig. 3.**
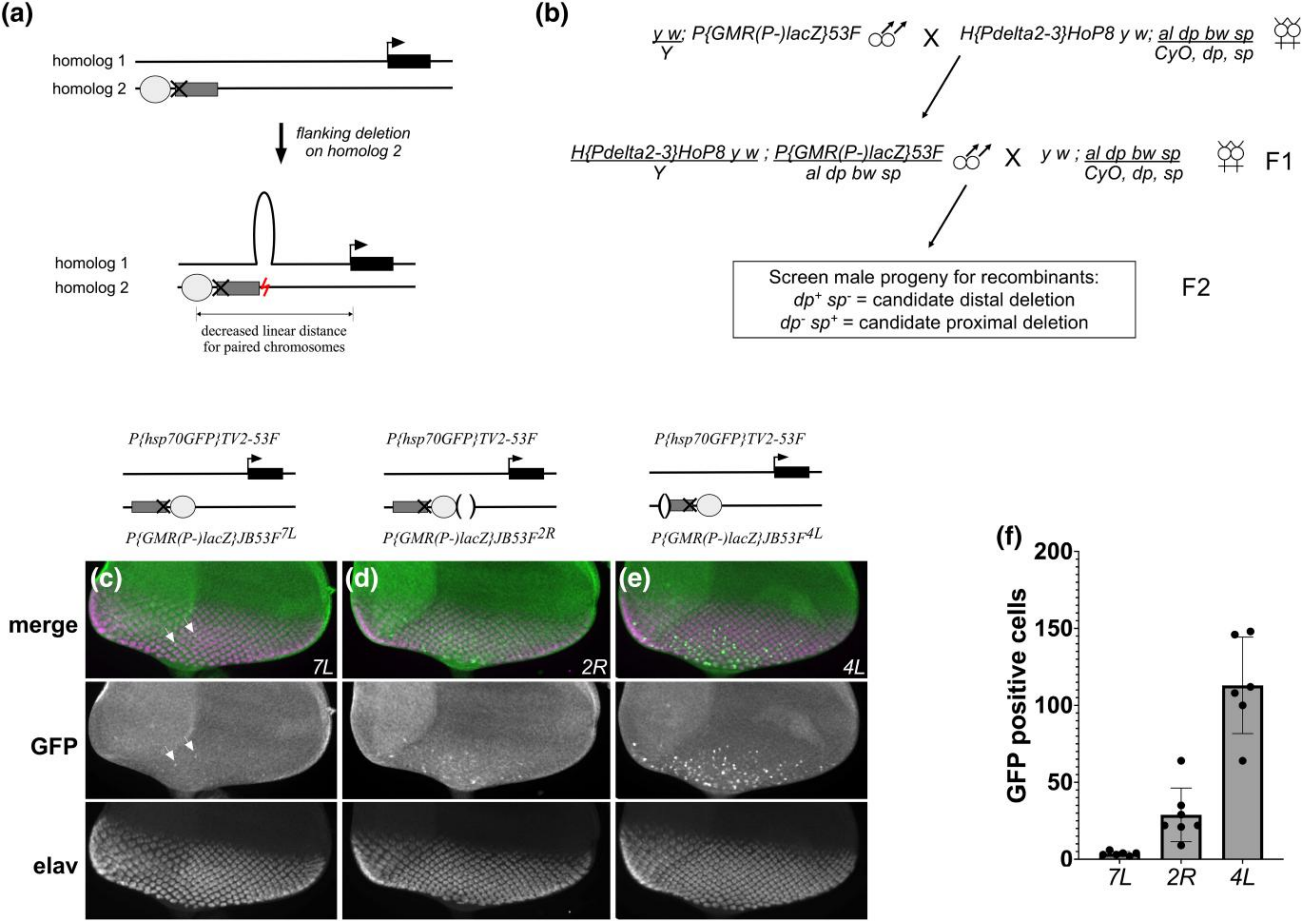
Deletions flanking a transgenic insertion can augment transvection between nonallelic positions. a) Schematic showing theoretical changes to paired chromosomes when one homolog carries a deletion located between 2 nonallelic positions. Subsequent “looping out” of unpaired DNA on the intact homolog results in a decreased linear distance of paired DNA between the 2 sites. b) Genetic scheme for generating deletions flanking *P{GMR(P-)lacZ}JB53F*. See *Materials and Methods* for a detailed description of the screen. c–e) Transvection between *P{GMR(P-)lacZ}JB53F* with flanking deletions and *P{hsp70GFP}TV2-53F* on an intact chromosome. Columns of images show merged or individual channels for staining with anti-GFP or anti-Elav; schematics above each image set show configurations of transgenes. c) Control *P{GMR(P-)lacZ}JB53F^7L^* carrying no deletion. Arrows indicate GFP-positive cells. d) *P{GMR(P-)lacZ}JB53F^2R^* with an 813 bp distal deletion. e) *P{GMR(P-)lacZ}JB53F^4L^* with an approximately 1 kb deletion internal to the transgene that includes the entire 5′ P-element end. f) Quantification of GFP-positive cells per disc for each of the genotypes presented in c–e). Each genotype carrying a deletion (*2R*, *4L*) is significantly different from the control (*P* < 0.05, Mann–Whitney *U* test).

A screen to generate flanking deletions around the Promoterless *GMR* P-element insertion near *GstS1* is outlined in Fig. 3b. Male flies carrying *P{GMR(P-)lacZ}JB53F* were crossed to virgin females of genotype *H{Pdelta2-3}HoP8 y w; al dp bw sp/CyO, dp, sp* to generate F1 male progeny of genotype *H{Pdelta2-3}HoP8 y w/Y; P{GMR(P-)lacZ}JB53F*/*al dp bw sp*. In the germlines of these flies, aberrant mobilization of the P-element at 53F can create Chromosome II recombination events at the site of the P-element that may also generate a small flanking deletion or duplication (Preston and Engels 1996); since the transgene is located between *dp* and *bw* on the second chromosome map, proximal deletions would be associated with recombinant chromosomes of genotype *al dp P{GMR(P-)lacZ}JB53F bw*^*+*^*sp^+^*, whereas distal deletions (in the direction of the Enhancerless GFP construct near *CG46491*) would be associated with recombinant chromosomes of genotype *al^+^ dp^+^ P{GMR(P-)lacZ}JB53F bw sp*. To screen for recombinant chromosomes, F1 males were crossed to virgin females carrying a *CyO* balancer marked with *dp* and *sp* mutations, allowing F2 progeny to be scored for at least one marker to the left and to the right of the P-element insertion. Only males were scored in the F2 to ensure loss of the P-element transposase that was carried on the X chromosomes of F1 males.

Approximately 2050 F2 males were scored, and 20 candidate recombinants were identified. Fifteen of these were successfully placed into stock by crossing to virgin females carrying a *CyO* balancer; of these, 9 were candidates for distal deletions, and 6 were candidates for proximal deletions based on flanking markers. Several candidates were tested for the presence of the Promoterless GMR P-element and for flanking deletions using primers GstS1_1, GstS1_2, GstS1_2_Ex1, Pend, RNXG9, Pry4, and Sp1 (see Supplementary Fig. 1 and Table 1 for primer locations and sequences). For *P{GMR(P-)lacZ}JB53F^4L^*, a proximal deletion candidate, sequencing of a GstS1_1/RNXG9 PCR fragment showed that it carries a 1,017 bp deletion that extends from a position 15 bp into the 5′ P end at the proximal end to a position 110 bp downstream of the transgenic AAUAAA polyadenylation signal at the distal end, thereby removing all but the terminal-most 15 bp of the 5′ P end in addition to the proximal attR recombination sequences. For *P{GMR(P-)lacZ}JB53F^7L^*, also a proximal deletion candidate, sequencing of a GstS1_1/Sp1 PCR product showed that it carries a small 30 bp tandem duplication of the 5′ P end inverse terminal repeat, but no other structural changes. For *P{GMR(P-)lacZ}JB53F^2R^*, a distal candidate, sequencing of a Pry4/GstS1_2_EX1 PCR product shows a deletion beginning 19 bp into the 3′ P end at the proximal end to a position 813 bp distal to the P insertion.

### Staining, microscopy, imaging, and scoring

Third instar eye-antennal discs were dissected, fixed, and stained with antibodies as previously described (Blick *et al*. 2016) using polyclonal rabbit anti-GFP (Invitrogen) diluted 1:2,000, mouse monoclonal anti-Elav [9F8A9, Developmental Studies Hybridoma Bank (DSHB)] diluted 1:400, mouse monoclonal antibeta-galactosidase (40-1a, DSHB) diluted 1:110, goat antirabbit AlexaFluor-488 secondary antibodies (Invitrogen) diluted 1:2,000, and/or goat anti-mouse Cy3 secondary antibodies (Jackson Immunoresearch) diluted 1:250. Stained discs were mounted in Fluoromount G with DAPI (Affymetrix) and visualized using a Zeiss Axio Imager.A2 epifluorescence microscope and AxioCam Mrm camera with Zen Lite software, with the exception of high magnification views of discs shown in Fig. 2, which were imaged using a Zeiss Axioplan 2 microscope with a 510 Meta confocal laser scanning system, with optical sections collected at 0.7-mm increments. Confocal images were processed in FIJI image analysis software (Schindelin *et al*. 2012) to combine *z*-stacks into a single flat image (using max intensity) for publication.

Counts of GFP-positive cells presented in Fig. 3 were determined from images of whole eye-antennal discs. To assess *mini-white* eye pigmentation, adult male flies of each genotype were collected within 8 h of eclosion and aged 5 days prior to scoring. Fly eyes were imaged under consistent light and exposure levels using a Canon EOS Rebel Tli digital camera mounted on a Leica MZ7.5 stereomicroscope using EOS Utility Imaging Software (Canon Inc.). At least 10 flies of each genotype were examined and scored separately by at least 2 scorers. Note that scoring was performed at the microscope and not from digital images.

## Results

We previously incorporated transgenes carrying fluorescent reporters that mimic Enhancerless and Promoterless alleles of classical transvection systems into several locations in the *Drosophila* genome (Bateman *et al*. 2012; King *et al*. 2019). Specifically, these reporters carry a coding region for a reporter gene (*lacZ* or *GFP*), and either the eye-specific synthetic enhancer *GMR* (Moses and Rubin 1991) or the minimal core promoter of the *hsp70* gene. When Enhancerless and Promoterless constructs are placed at allelic positions on homologous chromosomes within the same organism, enhancer action in *trans* can be observed via GFP fluorescence within differentiated photoreceptors of third instar larval eye discs (Fig. 1a–c).

By chance, 2 of our insertions are separated by only 2.6 kb between the *GstS1* and *CG46491* genes within polytene band 53F8 on chromosome arm 2R (Fig. 1b). Hi-C analysis of third instar eye-antennal discs supports that the 2 insertions are within the same Topologically Associated Domain (TAD) (Viets *et al*. 2019). To address whether transvection can be supported between transgenes at these nearby but nonallelic positions, we crossed flies carrying an Enhancerless construct at one location and a Promoterless construct at the other location and assessed fluorescence in third instar larval eye discs in the resulting progeny. In control crosses where Enhancerless and Promoterless transgenes are at allelic positions, GFP positive cells are apparent in the majority of mature ommatidial clusters, consistent with previous observations (Fig. 2a and b; Bateman *et al*. 2012; King *et al*. 2019). When the Enhancerless construct near *CG46491* is paired with the Promoterless construct near *GstS1*, GFP positive retinal cells are observed in all discs examined (*n* = 7), with fewer positive cells relative to the control discs carrying constructs in allelic positions (Fig. 2c). In the reverse configuration with an Enhancerless construct near *GstS1* and a Promoterless construct near *CG46491*, very few GFP positive cells are observed (Fig. 2d); however, at least 1 GFP positive cell was observed in each disc examined (*n* = 7), whereas negative control discs carrying Enhancerless or Promoterless constructs alone show no evidence of GFP expression (Bateman *et al*. 2012; King *et al*. 2019). Thus, enhancer action in *trans* is supported between transgenic insertions separated by 2.6 kb, with differences in the levels of activation depending on the positions of the enhancer and promoter.

### A deletion on one homolog augments transvection between nonallelic sites

We next asked whether transvection between nonallelic sites could be improved by decreasing the distance between the positions of the transgenic insertions on one of the homologous chromosomes, which we predicted would alter the pairing configuration of the locus to “loop out” the unpaired portion of the unaffected chromosome and thereby bring the enhancer and promoter into closer proximity (Fig. 3a). To accomplish this, we used P-element-induced male recombination to generate chromosomal deletions flanking the Promoterless construct near the *GstS1* gene (Preston and Engels 1996). In this method, a P-element is given the opportunity to remobilize by introducing the P transposase via genetic cross. In some instances, an aberrant remobilization event can create a recombinant chromosome carrying a small deletion adjacent to the P-element that otherwise remains in its original position; these events can be easily detected by screening for recombination events in the male germline where conventional meiotic crossing over does not take place (Preston and Engels 1996; Duttaroy 2002).

We screened 2,050 male progeny according to the cross scheme in Fig. 3b and identified 20 putative recombinants around the Promoterless construct near *GstS1*, a rate of 0.1% that is consistent with previous observations (Preston and Engels 1996; see *Materials and Methods*). Subsequent analysis showed that one line, *P{GMR(P-)lacZ}JB53F^2R^*, carried an approximately 800 bp deletion of genomic DNA immediately distal to the P-element, which meets our criteria of reducing the distance between the transgenic insertion sites on one homolog. A second recombinant line, *P{GMR(P-)lacZ}JB53F^4L^*, carried an approximately 1 kb deletion that removes the 5′ P-element end, with no changes to flanking genomic DNA. Finally, *P{GMR(P-)lacZ}JB53F^7L^* showed a small 30 bp duplication of the 5′ P-element terminal repeat but no major flanking deletions, and was used as a control.

We crossed each of the 3 recombinant lines to the Enhancerless construct near *CG46491* and assessed enhancer action in *trans* in larval eye discs. The recombinant control line with no deletions, *P{GMR(P-)lacZ}JB53F^7L^* (Fig. 3c), appeared qualitatively indistinguishable from the original *P{GMR(P-)lacZ}JB53F* construct that lacks structural changes (Fig. 2d), with very few GFP positive cells in each disc. In contrast, the recombinant line with the 800 bp distal deletion, *P{GMR(P-)lacZ}JB53F^2R^* (Fig. 3d), showed more GFP positive cells in each disc, indicating that the presence of the deletion augmented the capacity of *GMR* to act in *trans* from a nonallelic site. Surprisingly, the recombinant carrying a deletion of the 5′ P-element end, *P{GMR(P-)lacZ}JB53F^4L^* (Fig. 3e), showed a more dramatic increase in GFP expression, even though the deletion was on the opposite side of the Promoterless transgene relative to the Enhancerless transgene. To quantify these differences, we scored the number of GFP positive cells per disc for each genotype (Fig. 3f), which showed a significant increase in the number of GFP-positive cells per disc for *P{GMR(P-)lacZ}JB53F^2R^* (28.8 ±17.4 (mean ±standard deviation), *n* = 7 discs; *P* = 0.001, Mann–Whitney *U* test) and for *P{GMR(P-)lacZ}JB53F^4L^* (113.0 ±31.3, *n* = 6 discs; *P* = 0.002) relative to control *P{GMR(P-)lacZ}JB53F^7L^* discs (3.6 ±1.4, *n* = 6 discs). In sum, our data support that enhancer action in *trans* between nearby nonalleleic positions can be improved by decreasing the distance between the 2 locations on one of the homologous chromosomes, and that sequences within the 5′ P-element end may be detrimental to enhancer action in *trans*.

### Parameters additional to linear distance impact transvection between nonallelic sites

To further explore the potential for transvection between nearby nonallelic positions, we sought other transgenes carrying Enhancerless reporters that could be assessed for activation by *GMR*. To this end, several large-scale screens have generated thousands of insertions of P-elements and other transposons marked with *mini-white* (Bier *et al*. 1989; Huet *et al*. 2002; Bellen *et al*. 2004, 2011; Thibault *et al*. 2004). The *mini-white* reporter is essentially an Enhancerless transgene that relies on the local chromatin environment to permit transcription and subsequent eye color pigmentation, ranging from yellow (low transcription) to red (high transcription) when assessed in an otherwise *white*^*−*^ background (Pirrotta 1988). We first determined whether *mini-white* could be activated by *GMR* from an allelic position in *trans* by crossing the Promoterless *GMR* transgene near *GstS1* to its parent landing site *P{attP.w^+^.attP}JB53F*, which is marked with *mini-white* (Bateman and Wu 2008). In the absence of *GMR* in *trans*, *mini-white* expression from *P{attP.w^+^.attP}JB53F* results in an orange eye color, reflecting an intermediate level of transcription. When *GMR* is placed in *trans* at the allelic position, the resulting eye color is a robust red, supporting that *mini-white* transcription can be augmented by *GMR* enhancer action in *trans* (Supplementary Fig. 2).

**Fig. 4.**
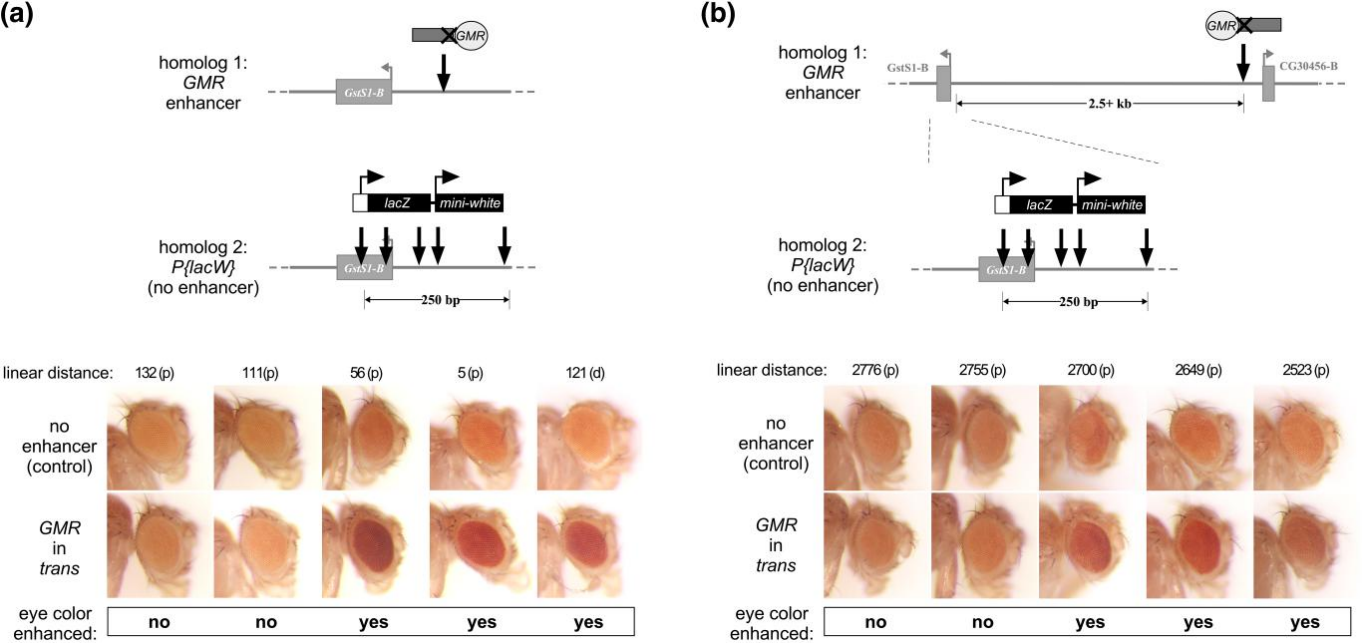
*GMR* action in *trans* on nearby nonallelic *mini-white* insertions at the 53F locus. Each schematic shows the position of a transgene carrying *GMR* on homolog 1 and the positions of 5 different *P{lacW}* insertions near *GstS1-RB* on homolog 2. Images show adult eye pigmentation for flies where *GMR* is carried on homolog 1 (bottom row) and for control flies where homolog 1 does not carry a transgene (top row). The linear distance in base pairs from the *P{lacW}* insertion site to that of the *GMR* transgene [either proximal (p) or distal (d)] is indicated above each image set. Qualitative assessment of enhancement of pigmentation in the presence of *GMR* relative to control is indicated below the images for each *P{lacW}* insertion. a) *GMR* transgene *P{GMR(P-)lacZ}JB53F* inserted near *GstS1-RB*; b) *GMR* transgene *P{GMR(P-)GFP}TV2-53F* inserted near *CG46491-RB*.

We then searched the *Drosophila* genome annotation for transgenes carrying *mini-white* that had been inserted near *GstS1* in polytene band 53F. Fortuitously, the *GstS1* gene is a P-element hotspot, and many insertions have mapped near the promoter of the *GstS1-RB* transcript (Wangler *et al*. 2015). To more effectively compare transgenes inserted in different locations, we identified 5 nearby insertions of a single transgene type, *P{lacW}* (Table 1; Bier *et al*. 1989), for which stocks were available. All 5 insertions were mapped within 250 bp of one another surrounding the *GstS1-B* transcriptional start site, and all were inserted in the same orientation on the chromosome (Bellen *et al*. 2004; Fig. 4a).

We first crossed each *P{lacW}* line to the Promoterless *GMR* construct near *GstS1* (Fig. 4a). Surprisingly, only 3 of the 5 *P{lacW}* insertions showed elevated *mini-white* expression relative to control flies without *GMR* in *trans*, even though the largest distance between *P{lacW}* insertions on one chromosome and the Promoterless *GMR* insertion on the homolog was just 132 bp. Notably, the 2 *P{lacW}* insertions that failed to show evidence of transvection were both inserted in the 5′ UTR of the *GstS1-B* transcript, whereas the 3 insertions with clear enhancement by *GMR* were inserted in upstream noncoding DNA (Fig. 4a). We then crossed the 5 *P{lacW}* insertions to the Promoterless *GMR* construct inserted near *CG46491* approximately 2.5 kb away (Fig. 4b). We observed an identical pattern of enhancement as in the previous experiment, with the 3 *P{lacW}* insertions in noncoding DNA showing enhancement of *mini-white* relative to controls lacking *GMR*, and the 2 insertions in the *GstS1-RB* UTR showing no evidence of enhancement. Our data therefore show that the linear distance between transgenic insertions is not the sole predictor of whether transvection can be supported between them.

**Fig. 5.**
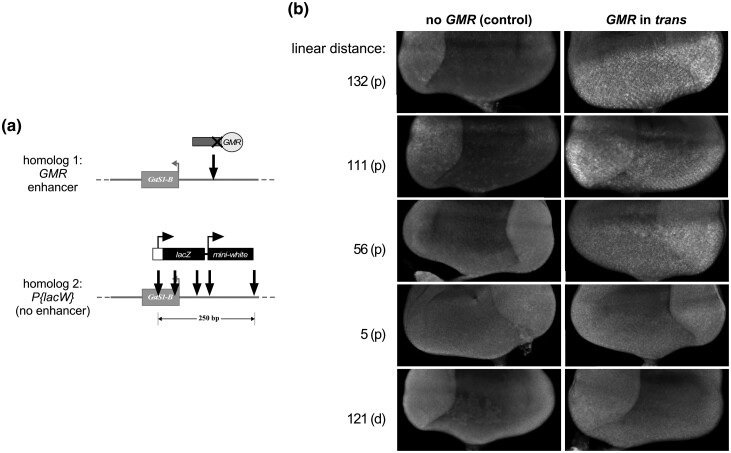
*GMR* action in *trans* on nearby nonallelic *lacZ* insertions differs from *mini-white* activation at the same positions. a) Schematic showing *GMR* transgene *P{GMR(P-)lacZ}JB53F* inserted near *GstS1-RB* on homolog 1 and 5 *P{lacW}* insertions near *GstS1-RB* on homolog 2. b) Representative third instar eye discs stained with antibeta-galactosidase antibodies where *GMR* is carried on homolog 1 (right column) and for control flies where homolog 1 does not carry a transgene (left column). The linear distance in base pairs from the *P{lacW}* insertion site to that of the *GMR* transgene [proximal (p) or distal (d)] is indicated to the left of each image set.

Each *P{lacW}* insertion carries a second transgene encoding Beta-galactosidase (from the *Escherichia coli lacZ* gene) and driven from the endogenous P-element promoter, which was originally intended as an “enhancer trap” to report the activity of endogenous enhancers near the point of insertion (Bier *et al*. 1989). As a complementary test of *GMR's* capacity to act in *trans* on nearby nonallelic positions, we crossed each of the 5 *P{lacW}* lines to the Promoterless *GMR* transgene near *GstS1* and stained third instar larval eye discs using antibodies against Beta-galactosidase (Fig. 5a and b). In control discs lacking the *GMR* enhancer in *trans*, each *P{lacW}* insertion line showed similar levels of weak to undetectable Beta-galactosidase signal. Surprisingly, in the presence of *GMR* in *trans*, Beta-galactosidase staining showed a trend almost completely opposite to that shown by *mini-white* derived from the identical insertions; specifically, the 2 *P{lacW}* insertions in the 5′ UTR of *GstS1-B* now showed robust Beta-galactosidase staining, whereas the 2 insertions furthest upstream of *GstS1-B* showed little to no detectable change in Beta-galactosidase levels. The remaining *P{lacW}* insertion, positioned just upstream of *GstS1-B*, showed modest activation of *lacZ* in larval eye discs in addition to activation of *mini-white* in adult flies, thereby behaving differently from insertions of identical constructs located only 50 bp on either side. In sum, our observations indicate that the capacity to support transvection between nearby nonallelic positions does not simply scale with linear distance along the chromosome; rather, other parameters unique to each insertion site and transgene must also play a role in permitting transvection at detectable levels.

### Position effects impact transvection between nonallelic sites

**Fig. 6.**
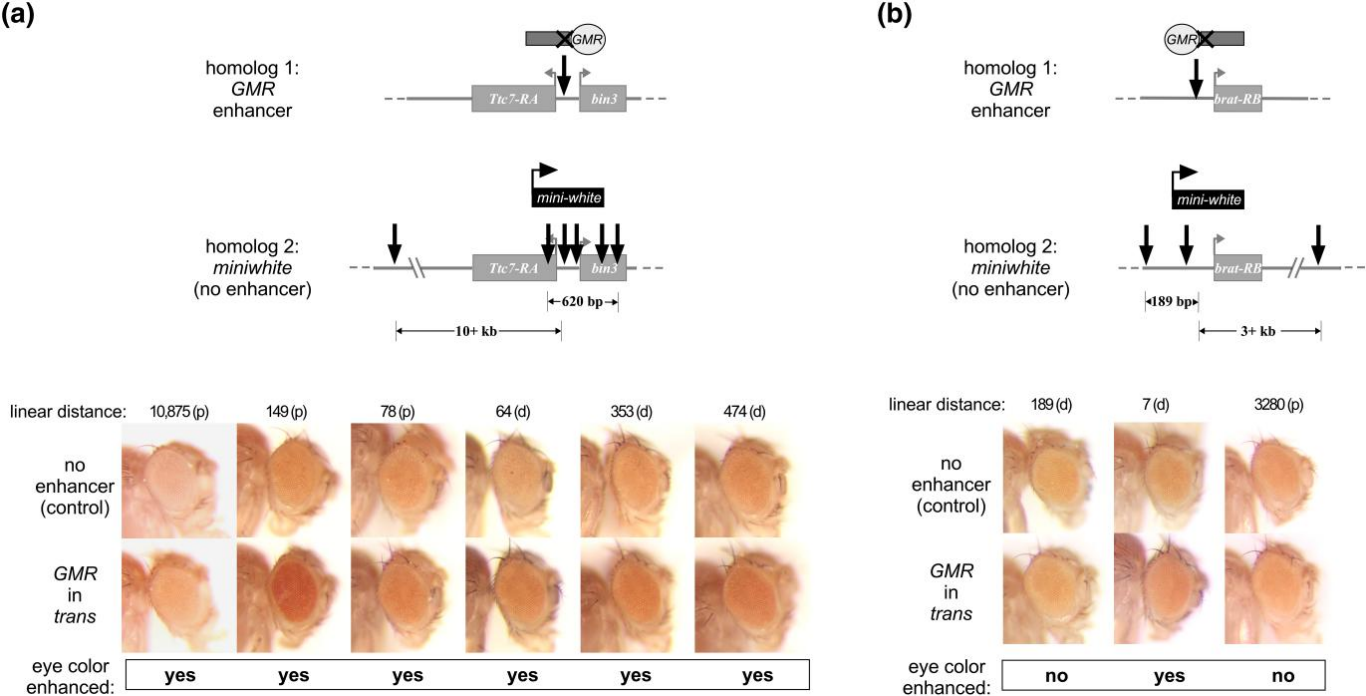
Position effects impact *GMR* action in *trans* on nonallelic *mini-white* insertions. Each schematic shows the position of a transgene carrying *GMR* on homolog 1 and the relative positions of several insertions carrying *mini-white* on homolog 2. a) Insertions near *Ttc-RA* and *bin3* at polytene position 47A13. b) Insertions near *brat-RB* at polytene position 37C5. RNA-seq shows that each of *Ttc7*, *bin3*, and *brat* is endogenously expressed in wild-type eye-antennal discs (Potier *et al*. 2014). Images show adult eyes for flies where *GMR* is carried on homolog 1 (bottom row) and for control flies where homolog 1 does not carry a transgene (top row), with the linear distance in base pairs from the *mini-white* insertion to that of the *GMR* transgene indicated above each image set. Qualitative assessment of enhancement of pigmentation in the presence of *GMR* relative to control is indicated below the images. Eye pigmentation was scored at the microscope by at least 2 different scorers for each *mini-white* insertion.

Finally, we asked whether transvection between nearby nonallelic sites can be supported at other genomic locations. We previously characterized *GMR* enhancer action in *trans* between allelic transgenic insertions at additional locations, including one upstream of the *Ttc7-RA* transcript in polytene band 42A13 and another upstream of the *brat-RB* transcript in polytene band 37C5 (King *et al*. 2019). For the *Ttc7* gene region, we found existing stocks for 6 transgenic insertions that were marked with *mini-white* and inserted relatively close to the *Ttc7-RA* transcription start site (Fig. 6a, Table 1; Bier *et al*. 1989; Huet *et al*. 2002; Bellen *et al*. 2004, 2011; Thibault *et al*. 2004). When flies carrying each *mini-white* insertion were crossed to the Promoterless *GMR* construct upstream of *Ttc7*, we observed an increase in the pigmentation of the adult eye relative to control flies lacking the *GMR* enhancer in *trans*; although the change in eye color was subtle in some cases, it was consistently observed over many age- and sex-controlled adults, demonstrating that transvection between the nonallelic sites occurred for each combination (Fig. 6a). Notably, the largest distance from the insertion site of the *mini-white* transgene to that of the Promoterless *GMR* transgene was 10.8 kb. In contrast, for the *brat* gene region, we found just 3 suitable stocks carrying *mini-white* insertions near the *brat-RB* transcription start site (Fig. 6b, Table 1). At this locus, only the *mini-white* transgene nearest (7 bp distal) to the insertion site of the Promoterless *GMR* construct showed a mild but consistent increase in eye pigmentation relative to control flies, whereas inserts that were 189 bp distal or 3.3 kb proximal to the GMR insertion site showed no evidence of transvection (Fig. 6b). In sum, our data show that enhancer action in *trans* between transgenes at nonallelic positions is possible for distances of at least 10.8 kb, but factors including position effects and transgene identity have a major influence on whether transvection will be supported.

## Discussion

Transvection in *Drosophila* relies on intimate pairing of homologous chromosomes so that regulatory elements can communicate in *trans*. In this study, we sought to better understand the pairing requirements for transvection by testing the capacity of enhancers and promoters to communicate in *trans* between nearby but nonallelic transgenic insertions. Our observations show that enhancer action in *trans* can indeed occur between some nonallelic insertions that are separated by a linear distance of at least 10 kb. However, our data also demonstrate a lack of transvection between some transgenes that are inserted at positions separated by only tens of base pairs, indicating that factors beyond the linear distance separating insertion sites likely play an important role in determining whether transvection between nonalleleic positions can be supported.

At the 53F8 locus, our data show that transgenic insertions separated by 2.6 kb can support transvection. Curiously, our data showed more robust enhancer action in *trans* by *GMR* when the enhancer was located near the *GstS1* gene and the *hsp70* promoter and *GFP* coding region were located near the *CG46491* relative to the reversed configuration. While this was not anticipated, it is possible that the chromatin environment upstream of *GstS1* is more favorable for transcription, or that subtle differences between the 2 types of transgenes used have an unknown influence on transcriptional efficiency. Note that our method of examining GFP fluorescence reflects the total output of transcription and translation resulting from transvection, and therefore does not directly inform our understanding of transcriptional dynamics of transvection as has been described elsewhere (Lim *et al*. 2018).

We predicted that a deletion of genomic DNA adjacent to one of the transgenic insertions would increase the efficiency of transvection between nonallelic sites by changing the local chromatin topology (Fig. 3a). In support of this prediction, an approximately 800 bp deletion flanking the promoterless *GMR* insert near *GstS1* increased the number of GFP-positive cells relative to a transgene lacking a deletion when each was placed in *trans* to the *hsp70-GFP* transgene near the *CG46491* gene. According to our model, the increased enhancer action in *trans* in the presence of the deletion is due to the unpaired DNA of the intact chromosome being “looped out” in this configuration, leading to an effectively reduced linear distance of paired chromatin between the insertions that could increase the probability of productive interactions between enhancer and promoter. While this model is supported by the data, we cannot exclude the possibility that some other aspect of the altered pairing topology is relevant to the observed change in transvection, or that the deletion in our experiment removed a DNA sequence that interferes with transvection independently of local chromatin folding. In our screen for flanking deletions, we also uncovered a deletion of sequences within the 5′ P-element end of the Promoterless transgene near *GstS1*, which dramatically increased the capacity of *GMR* to act in *trans* relative to the transgene without a deletion. Interestingly, a recent quantitative assessment of enhancer action in *trans* by *GMR* at multiple positions in the genome showed that an outlier line with an extraordinarily high capacity for transvection also carried a deletion in the 5′ P-element end (King *et al*. 2019). While this may be purely coincidental, Fujioka *et al*. (2021) recently showed that a promoter in the 5′ P-element end can be activated by transgenic enhancers in *cis*, which can result in reduced transcriptional output from the transgene depending on the orientation and configuration of transgenic elements. It is therefore possible that the presence of a nearby 5′ P-element end is detrimental to enhancer action in *trans*, and studies based on P-element transgenes may underestimate the strength of transvection.

We were further surprised by our analysis of *GMR* action in *trans* using *P{lacW}* insertions near *GstS1*, where differences in the activation of *mini-white* in *trans* differed dramatically among 5 lines with insertions that were separated by only 250 bp. It is unlikely that these differences are due simply to changes in linear distance between the different *P{lacW}* insertions and the position of the *GMR* transgene since the same pattern was seen when using either the Promoterless *GMR* construct near *GstS1* (within hundreds of bp from the *P{lacW}* insertions), or the Promoterless *GMR* construct near *CG46491* (approximately 2.5 kb away). Moreover, analysis of beta-galactosidase production resulting from *lacZ* transcription from the same *P{lacW}* insertions showed a remarkably different pattern, with robust staining observed for the lines that showed little to no evidence of *mini-white* activation. Although puzzling, these data may result from competition and/or interference among the promoters at the 53F8 locus. For example, for each of the *P{lacW}* insertions, transcription of *mini-white* occurs on the opposite strand relative to *GstS1*; with this configuration, transcriptional interference between the *mini-white* and *GstS1* promoters could prevent robust activation of *mini-white* for the *P{lacW}* insertions in the *GstS1-RB* 5′ UTR, whereas interference would not be predicted for insertions upstream of the *GstS1-RB* TSS. Furthermore, the *lacZ* transgene carried by *P{lacW}* insertions in the *GstS1-RB* 5′ UTR is positioned downstream of the *mini-white* transcription unit relative to the *GstS1-RB* promoter (Fig. 5). As such, any transcription of *mini-white* would be predicted to protect *lacZ* from transcriptional interference with *GstS1*, permitting *lacZ* transcription and subsequent beta-galactosidase production. In contrast, for the *P{lacW}* inserts upstream of the *GstS1-RB* TSS where transcriptional interference would not be predicted to occur, differences between the transgenic promoters that drive *lacZ* and *mini-white* may lead to favorable expression of the latter relative to the former. Specifically, *lacZ* is driven by the endogenous P-element promoter (Bier *et al*. 1989), which encodes only an *Initiator* (*Inr*) core promoter element and has previously been demonstrated to be a poor competitive target for enhancers acting in *trans* (Hodgetts and O’Keefe 2001; Mellert and Truman 2012). Conversely, the *white* promoter driving *mini-white* has both *Downstream Promoter Element* (*DPE*) and *Inr* core promoter elements, and prior analysis of transvection at the *yellow* locus has shown that it is a favorable target in *trans* (Kutach and Kadonaga 2000; Morris *et al*. 2004; Lee and Wu 2006). Ultimately, our observations highlight the potential complexity of outcomes when enhancers choose among promoter targets in *cis* and in *trans*.

Our data further show that transvection between nonallelic transgenic insertions is influenced by position effects, where an insertion near *Ttc7* supported *GMR* activation of *mini-white* in *trans* from more than 10 kb away, whereas an identical transgene inserted near *brat* showed no evidence of *trans*-activation of a *mini-white* insertion <200 bp away. Several potential mechanisms could account for these differences. For example, the chromatin environment near *Ttc7* could simply be permissive for higher levels of transcription; in support of this, King *et al*. (2019) showed that *GMR* activation of *hsp70-GFP* in *cis* results in a roughly 2-fold increase in fluorescence for the insertion near *Ttc7* relative to the insertion near *brat*. Alternatively, Hi-C strategies have recently shown that the “tightness” of pairing varies across the genome, with some loci holding homologous chromosomes in tight register, and others allowing looser “sloppy” pairing (AlHaj Abed *et al*. 2019; Erceg *et al*. 2019). It could therefore be that the *Ttc7* locus reflects a looser pairing state that permits higher levels of contact between nearby nonallelic regions of homologous chromosomes, whereas the *brat* locus may be held in a tighter paired state where *trans*-interactions are more restricted. Other analyses of transvection using transgenic insertions have shown that sequences such as insulators and PREs can facilitate interactions between insertions at nonallelic positions, possibly due to clustering of these elements into nuclear bodies (Sigrist and Pirrotta 1997; Muller *et al*. 1999; Kassis 2002; Bantignies *et al*. 2003; Pirrotta and Li 2012). Thus, position effects on transvection could also reflect the presence or absence of nearby insulator and PRE sequences. Ultimately, it is likely that a complex interplay of chromatin structure and topology influenced by local genetic elements will determine the capacity for productive *trans*-interactions between nonallelic sites over varying linear distances.

## Supplementary Material

jkad255_Supplementary_Data

## Data Availability

The authors affirm that all data necessary for confirming the conclusions of the article are present within the article, figures, and tables. Stocks are available upon request. Supplemental material available at G3 online.
