# Supplementary material for: Transvection between nonallelic genomic positions in Drosophila
Source: G3 (Bethesda). 2023 Nov 9;14(2):jkad255. doi: 10.1093/g3journal/jkad255 (PMC10849331; doi:10.1093/g3journal/jkad255)
Supplement: jkad255_Supplementary_Data [file jkad255_supplementary_data.docx]

**Supplemental Table 1.** Primers used in this study.

| **Primer name** | **Sequence** |
| --- | --- |
| GstS1_1 | TCTGGGACCCCTAGCCCCGA |
| GstS1_2 | GGCACCGCCAGTCTGGATCG |
| Pend | GACGGGACCACCTTATGTTA |
| Sp1 | ACACAACCTTTCCTCTCAACAA |
| Plac1 | CACCCCAAGGCTCTGCTCCCACAAT |
| Plac4 | ACTGTGCGTTAGGTCCTGTTCATTGTT |
| RNXG9 | GTGGTTTGTCCAAACTCATCAA |
| Pry4 | CAATCATATCGCTGTCTCACTCA |
| SPEP1 | GACACTCAGAATACTATT |
| GstS1_2_EX1 | GGGAGTCCCATGGCTGCGAA |
| GstS1_2_EX2 | CCGCCGCTCATTCTTCGTGC |
| GstS1_2_EX3 | AACCAAAATCGAAGCTATGGGCGA |

**Supplemental Figure 1**


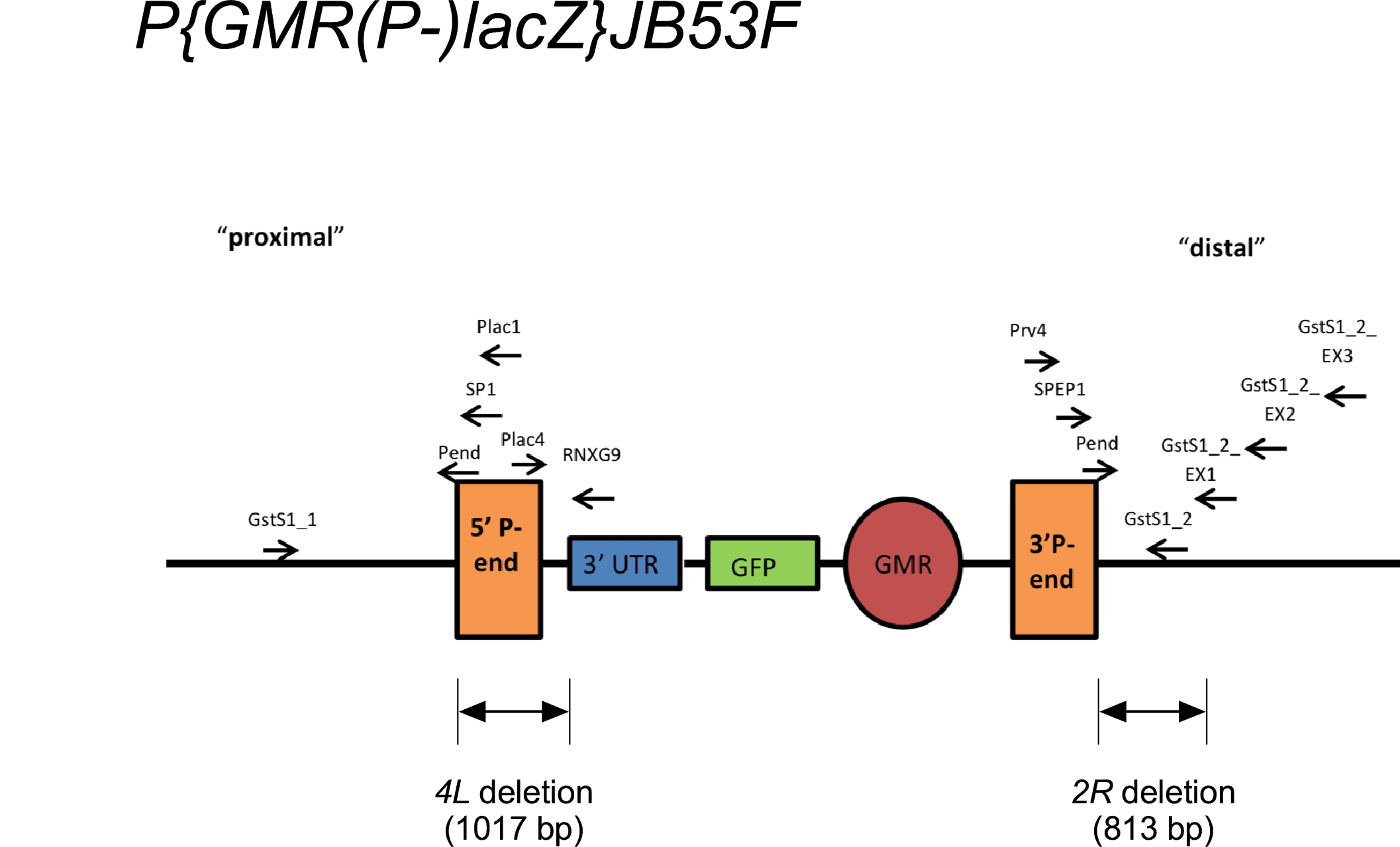


**Supplemental Figure 1.** Cartoon map of the *P{GMR(P-)lacZ}JB53F* insertion, showing the 4L and 7L deletions uncovered in the male recombination screen. Positions of primers used to analyze deletions are shown (see Supplemental Table 1 for primer sequences.)

**Supplemental Figure 2**


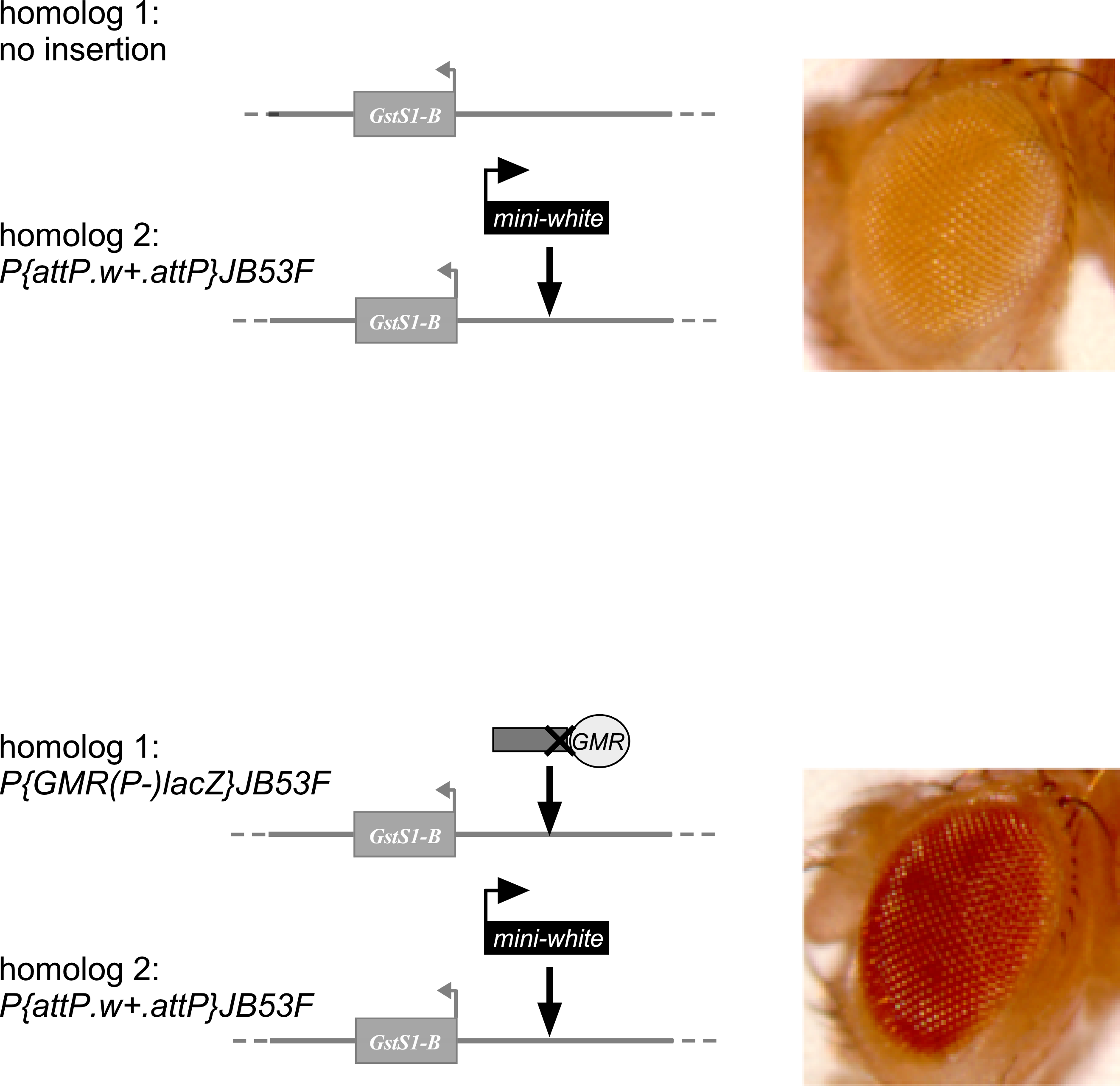


**Supplemental Figure 2.** *Mini-white* transcription is activated by *GMR* in *trans* at the *P{attP.w^+^.attP}JB53F* transgenic insertion site. Top, hemizygous insertion of *P{attP.w^+^.attP}JB53F* produces a yellow-orange eye, indicating low levels of *mini-white* transcription. Bottom, the *GMR* enhancer placed in *trans* to *mini-white* (at the identical insertion site) leads to a red eye, indicating an increase in *mini-white* transcription.
